# Retirement timing, living arrangements and mental health: a multi-method investigation using Survey of Health, Ageing and Retirement in Europe

**DOI:** 10.1017/S2045796026100833

**Published:** 2026-08-05

**Authors:** Jane Maddock, Cassandra Simmons, Jacques Wels, Praveetha Patalay

**Affiliations:** 1 University College London Division of Surgery & Interventional Science, UK; 2 Unit for Lifelong Health and Ageing at UCL, University College London, London, UK; 3 Health & Care, European Centre for Social Welfare Policy and Research, Austria; 4 Erasmus School of Health Policy and Management, Erasmus University Rotterdam, Netherlands; 5 Health & Society Research Unit, Université libre de Bruxelles, Belgium; 6 Centre for Longitudinal Studies, University College London, London, UK

**Keywords:** cohabitation status, health inequalities, mental health, retirement, triangulation

## Abstract

**Aims:**

The relationship between retirement and mental health remains unclear, with mixed evidence likely driven by methodological challenges and heterogeneity across population subgroups. This study explores the relationship between retirement, time since retirement and mental health, focusing on differences between those living alone and cohabiting.

**Methods:**

We used data from 10,698 participants (48,815 observations) in the Survey of Health, Ageing and Retirement in Europe, waves 1–8, spanning 20 countries and 16 years. Participants were included if they were employed at baseline and retired during follow-up. We applied a triangulated approach using (1) multilevel linear spline models to examine trajectories and (2) fixed-effects instrumental variable analyses (IV) using statutory retirement age as an instrument to establish causal effects. The outcome was depressive symptoms measured using the EURO-D scale (higher scores indicate more depressive symptoms).

**Results:**

Multilevel spline models showed minimal change in depressive symptoms before retirement. At retirement, a small decline in EURO-D scores was indicated (−0.07 points per year [95% CI: −0.13 to −0.0003]), followed by an increase starting two years post-retirement (0.09 per year, 95% CI: 0.02 to 0.16). IV analyses showed that retirement was associated with fewer depressive symptoms (−0.26, 95% CI: −0.45 to −0.07). Individuals living alone reported higher depressive symptoms, but cohabitation status did not moderate the relationship between retirement and mental health.

**Conclusions:**

Retirement is associated with a short-term reduction in depressive symptoms, but the effect diminishes over time. While individuals living alone consistently report higher depressive symptoms, cohabitation status does not moderate the retirement-mental health relationship.

## Introduction

A challenge for ageing populations and increasing life expectancies (United Nations, 2017) is ensuring that longer lives are spent in good health (Beard *et al.*, 2016). Maintaining good mental health in later life is important for achieving this goal. People undergo many life transitions that can impact their mental health (Kuh *et al.*, 2003), such as retirement. Retirement can involve loss of purpose, lower income, disruption of daily routines and diminished social connections (Kuhn, 2018; Odone *et al.*, 2021). Conversely, it may provide increased leisure time, relief from work-related stress and greater opportunities for social engagement (Kuhn, 2018; Odone *et al.*, 2021).

The extent to which retirement affects mental health remains inconclusive despite many empirical studies on this topic (Dave *et al.*, 2008; Jokela *et al.*, 2010; Coe and Zamarro, 2011; Behncke, 2012; Eibich, 2015; Belloni *et al.*, 2016; Fé and Hollingsworth, 2016; Heller-Sahlgren, 2017; Kolodziej and García-Gómez, 2019; Picchio and van Ours, 2020; Vo and Phu-Duyen, 2023). Generally, reviews and meta-analyses have found that retirement can positively affect mental health, although the findings vary (van der Heide *et al.*, 2013; Odone *et al.*, 2021; Garrouste and Perdrix, 2022; van Ours, 2022). This ambiguity may be partly due to methodological challenges in establishing causality and anticipatory effects, and heterogeneous effects among different populations and subgroups (Maddock and Wels, 2024).

The mental health consequences of retirement are likely to differ across population subgroups, and prior analyses focusing solely on overall effects may overlook important heterogeneity in these relationships (Maddock and Wels, 2024). One group that may be particularly vulnerable to mental health challenges during the transition to retirement is individuals living alone. Living alone is an objective indicator of social isolation (Holt-Lunstad *et al.*, 2015), and prior studies have demonstrated its association with poor mental health (Leigh-Hunt *et al.*, 2017). Retirement may result in a decreased social network size (Börsch-Supan and Schuth, 2014). Therefore, people who live alone as they transition from employment to retirement may be particularly vulnerable to experiencing mental health difficulties.

The methodological challenges in studying retirement and mental health also need to be addressed. Prior research using Survey of Health, Ageing and Retirement in Europe (SHARE) data suggests nonlinear patterns in mental health around retirement, with improvements shortly before and after retirement (Vo and Phu-Duyen, 2023). Meta-analytic evidence similarly indicates heterogeneous and time-varying effects (Odone *et al.*, 2021; van Ours, 2022). Applying a multilevel spline model will allow for the examination of how mental health changes as individuals transition into retirement (Steele, 2008). Using this approach, we can capture changes in mental health over time in relation to the duration since retirement, including possible anticipatory changes in mental health. However, despite careful adjustment of measured confounders, residual confounding due to, for example, unmeasured work-related stressors, or socioeconomic influences is possible. Applying an instrumental variable (IV) approach can overcome issues of residual confounding and reverse causality but is limited to examining the effect only for those whose retirement decision was induced by the instrument (Angrist *et al.*, 1996). These findings motivate our use of flexible spline models to capture potential nonlinear changes in trajectories before, during and after the retirement transition, combined with IV approaches to establish causal effects. Using a multi-method approach may provide a more nuanced insight into how retirement affects mental health. The multilevel model offers descriptive and inferential insights into changes over time, while the IV model strengthens causal inference.

Using data from European participants, our study explored the nuanced relationship between retirement, time since retirement and mental health, particularly distinguishing between individuals living alone and those cohabiting during this life transition. We applied a triangulated approach using two complementary analyses: multilevel spline models and fixed-effects IV analyses (Lawlor *et al.*, 2017). We address the following research questions with these approaches:

1. How does time since retirement influence changes in mental health, and does this relationship differ by cohabitation status?
2. What is the effect of retirement, as determined by statutory retirement age, on mental health, and how does this effect differ by cohabitation status?

## Methods

### Participants

The Survey of Health, Ageing and Retirement in Europe (SHARE), initiated in 2004/2005, is a biannual panel study providing information on health, socioeconomic and social circumstances from representative samples of individuals aged 50 years and over from 28 European countries and Israel (Börsch-Supan *et al.*, 2013; SHARE, 2022). Waves 3 (2008/2009) and 7 (2016/2017) include the ShareLife modules, which provide retrospective information on participants’ lives. Data are collected during face-to-face interviews using computer-assisted personal interviewing. We include data from waves 1 (2004/2005) to 8 (2019/2020). We exclude wave 9 to focus on the pre-COVID-19 period.

We included 48,815 observations from 10,698 participants (48% female) who were aged 50 years and older, were in employment or self-employed at their first interview and self-reported retired by their last interview and had measures on mental health from at least two waves. We excluded participants who had missing or implausible values for their reported retirement year (see flow chart in Supplementary Figure S1). Supplementary Table S1 provides a comparison of our analytical sample to the excluded SHARE population. Our analytical sample included participants from 20 countries (Austria, Belgium, Croatia, Czech Republic, Denmark, Estonia, France, Germany, Greece, Hungary, Israel, Italy, Luxembourg, Netherlands, Poland, Portugal, Slovenia, Spain, Sweden and Switzerland). We used SHARE release 9.0.0.

We used SHARE’s calibrated survey weights from participants’ baseline wave to address issues of unequal selection probabilities and unit non-response (Börsch-Supan *et al.*, 2013). We rescaled the weights to sum to our analytical sample size to preserve the calibration properties while maintaining the relative weighting structure and ensuring comparability across models. In addition, SHARE uses the hot-deck and fully conditional specification method to impute missing values due to item non-response within each wave. Complete details about this process are available in previous publications (Christelis, 2011; De Luca, 2018). SHARE provides multiple imputations, which we used for missing values for EURO-D, employment, years of education, number of children and number of chronic illness, where there is a small proportion of missing values (<5%). For household income, SHARE applies the fully conditional specification method since there is a larger proportion of missing values (>30%). We combined estimates across imputations using Rubin’s rules, implemented via Stata’s mi estimate command. We conducted sensitivity analyses using the complete cases (*n* = 7,645 participants after adjustment) and did not observe differences in our conclusions (data available on request).

### Mental health

The 12-item EURO-D scale was used to derive a depression symptoms scale. The EURO-D was originally developed from instruments on late-life depression used in European countries (Prince *et al.*, 2018; Fong and Chan, 2025). EURO-D contains the following items: depression, pessimism, suicidality, guilt, sleep, interest, irritability, appetite, fatigue, concentration, enjoyment and tearfulness (Mehrbrodt *et al.*, 2021). The score ranges from 0, indicating no depression symptoms, to 12, with a score of 4 or more indicating clinically significant depressive symptoms. We present results in both the original scale and standardised form (mean = 0 and SD = 1).

### Retirement

Participants self-reported their employment status at each wave. Response options were retired, employed or self-employed, unemployed, permanently ill or disabled, homemaker or other. We define retired as those who self-report retired and employed as self-report employed or self-employed. We consider the first report of retirement as a persistent state; individuals remain retired regardless of their subsequent reported employment state (<3.7% transitioned from retirement back into employment during the follow-up period but were retired by their last observation).

Participants could also report which year they retired. We calculated the duration of retirement by subtracting the reported retirement year from each interview year.

We used the ShareLife job panel dataset to extract country-, age-, gender- and year-specific statutory retirement ages (Brugiavini *et al.*, 2013; Antonova *et al.*, 2014) and mapped them to individuals within our analytical sample. Statutory retirement ages were based on information provided by the Mutual Information System on Social Protection, which provides information on social protection systems for European Union member states (Mutual Information System on Social Protection, 2024).

### Cohabitation status

We created a binary variable (0 = living alone, 1 = cohabiting when reporting initial retirement) based on participant reports of number of people living in their household. This single time-point measure captures cohabitation status at the time of first observed retirement, which is most relevant for our research questions and avoids bias, as later changes may mediate the relationship between retirement and mental health. Cohabitation was stable, with about 11% of participants changing status during follow-up.

### Covariates

Drawing from prior empirical studies, we incorporated variables that may influence both retirement and mental health. These included sex, age (in years, included as a continuous linear variable), years of education, total equivalised household income, number of children in the household, number of chronic conditions, and county of residence.

Education was measured using the International Standard Classification of Education and coded as the number of years in education (Mehrbrodt *et al.*, 2021). We derived equivalised total household income (reported in EUROS) to adjust household incomes to household composition based on the Organisation for Economic Co-operation and Development’s equivalence scale (Office for National Statistics, 2015). The number of children includes all natural, fostered, adopted and stepchildren that are still alive. The equivalised income was calculated as total household income/(1 + 0.5 × [number of adults − 1] + 0.3 × number of children), where adults are defined as household members aged 14 years or older. The number of chronic conditions is based on self-reported doctor-diagnosed conditions (see Supplementary file for details).

### Analyses

Baseline characteristics for the study participants are presented by cohabitation status as proportions and means and standard deviations.

We used linear multilevel spline models with three levels to address research question one. Multilevel models can account for the non-independence of observations in hierarchical structures. In our models, measurement occasions are at level 1, individuals are at level 2 and countries of residence are at level 3. We applied rescaled calibrated weights at level 1 as these weights were designed to account for individual selection and response. Stata’s mixed command models clustering of repeated observations within individuals and individuals within countries through the three-level random effects structure. Our main exposure of interest in these models is the time since retirement, centred at zero. We expect the association between time since retirement and EURO-D to be non-linear. Therefore, we fit piecewise polynomial functions, or splines, to these models (Howe *et al.*, 2016). We find the spline model to be a better fit than a linear model (*p* from likelihood-ratio test in unweighted data < 0.001). We selected our spline knot points based on prior research (Vo and Phu-Duyen, 2023) to be at the time of retirement, 2 years prior and 2 years post-retirement. To account for variability in how time since retirement influences depressive symptoms, we included random slopes for time since retirement and its splines at the individual level (level 2). We adjusted our models for age, sex, years of education, equivalised household income, number of children and number of chronic conditions. We stratified our analyses by cohabitation status in addition to including an interaction term between time since retirement and cohabitation status to explore whether there were differences between time since retirement and changes in mental health between those who cohabit or live alone when they retire. We also present the main models stratified by sex in the supplementary materials. Since our results may be sensitive to the selection of the spline knot points, we repeat these analyses using knot points defined at (1) the time of retirement, 1 year prior and 1 year post-retirement; (2) the time of retirement, 6 years prior and 6 years post-retirement; and (3) the time of retirement, 10 years prior and 10 years post-retirement.

We used fixed-effects IV analyses with cluster-robust standard errors clustered at the individual level to address the second research question. Fixed-effects models account for unobserved time-invariant differences, such as individual preferences about when to retire or long-term health characteristics. The IV approach uses an exogenous instrument to reduce bias from reverse causation and unmeasured confounders (Hernán and Robins, 2006). We first examined associations between retirement status (employed/self-employed vs retired) using a fixed-effects model adjusted for age, years of education, equivalised household income, number of children and number of chronic conditions.

We then applied the IV analyses. As with previous studies (Coe and Zamarro, 2011; Behncke, 2012; Belloni *et al.*, 2016; Heller-Sahlgren, 2017; Vo and Phu-Duyen, 2023), we used sex-, country- and year-specific statutory retirement age to define our instrument. We coded the instrument as one if age is equal to or greater than the statutory retirement age and zero otherwise. We then stratified the analyses by cohabitation status and included an interaction term in our IV models. We also present sex-stratified results in supplementary materials. The IV estimates represent the Local Average Treatment Effect (LATE), that is, the causal effect of retirement for compliers whose retirement decision was induced by reaching statutory retirement age. There are three key assumptions required for our instrument to be valid (Hernán and Robins, 2006; Davies *et al.*, 2013). First, the instrument must be strongly related to the likelihood of retirement. Second, the instrument must be independent of any confounding factors that could bias the relationship. Third, the instrument must not influence EURO-D scores directly, except through its effect on retirement. We report the partial F-statistic to examine the first assumption (Davies *et al.*, 2013). While the latter two cannot be proven, they can be falsified, which we explore using a series of regression models with robust standard errors. An additional assumption of monotonicity requires that reaching the statutory retirement age does not cause anyone to delay retirement. This is plausible given that statutory age represents eligibility for full pension benefits, creating financial incentives to retire. Finally, to provide additional validation for our instrument, we also conducted a falsification test using a negative control instrument. To do this, we redefined our IV to be statutory retirement age minus 7 years. Assuming our original instrument is valid, this negative control should show no effect on EURO-D scores, as any observed association would indicate spurious trends rather than true retirement effects.

## Results

Of the 10,698 participants (*n* = 48,815 observations) in our analytical sample, 11% had information from all time points (Table S2). Supplementary Table S3 outlines the participants and observations by country of residence.

The baseline characteristics of the sample by cohabitation status are displayed in Table 1. A total of 17% of participants were living alone during retirement and 65% of those living alone were female. While those living alone and females had higher (weighted) mean EURO-D scores (2.4 [95% CI: 2.2–2.5]; 2.5 [95% CI: 2.4–2.6]) compared with those cohabiting and males (1.9 [95% CI: 1.8–2.0]; 1.6 [95% CI: 1.5–1.7]), the association between cohabitation status and EURO-D did not vary by sex (*p* = 0.917). Table S4 in supplementary materials displays the weighted means and 95%CI of EURO-D by sex and cohabitation status.

**Table 1.** Baseline characteristics of study participants by cohabitation status at the time of retirement 1 long description.

	Cohabiting	Living alone	Total
	8,896 (83%)	1,802 (17%)	10,698
Age (years)	57 (4.3)	57 (4.7)	57 (4.4)
Gender			
Male	5,338 (61%)	802 (40%)	6,139 (57%)
Female	3,349 (39%)	1,209 (60%)	4,559 (43%)
EURO-D	1.88 (1.85)	2.37 (2.15)	1.98 (1.92)
Years of education	12.01 (4.19)	12.13 (4.29)	12.03 (4.21)
Equivalised household income	23,451 (27,885)	22,050 (19,688)	23,117 (26,167)
Number of chronic conditions	1.03 (1.08)	1.17 (1.14)	1.06 (1.09)
Number of children in household	2.14 (1.13)	1.44 (1.26)	2.01 (1.19)

Values are mean (SD) or *N* (%) and based on observed data using calibrated survey weights. EURO-D ranges from 0 to 12 where higher scores indicate more depressive symptoms.

### Research question one: how does time since retirement influence changes in mental health and does this relationship differ by cohabitation status?

**Figure 1.**
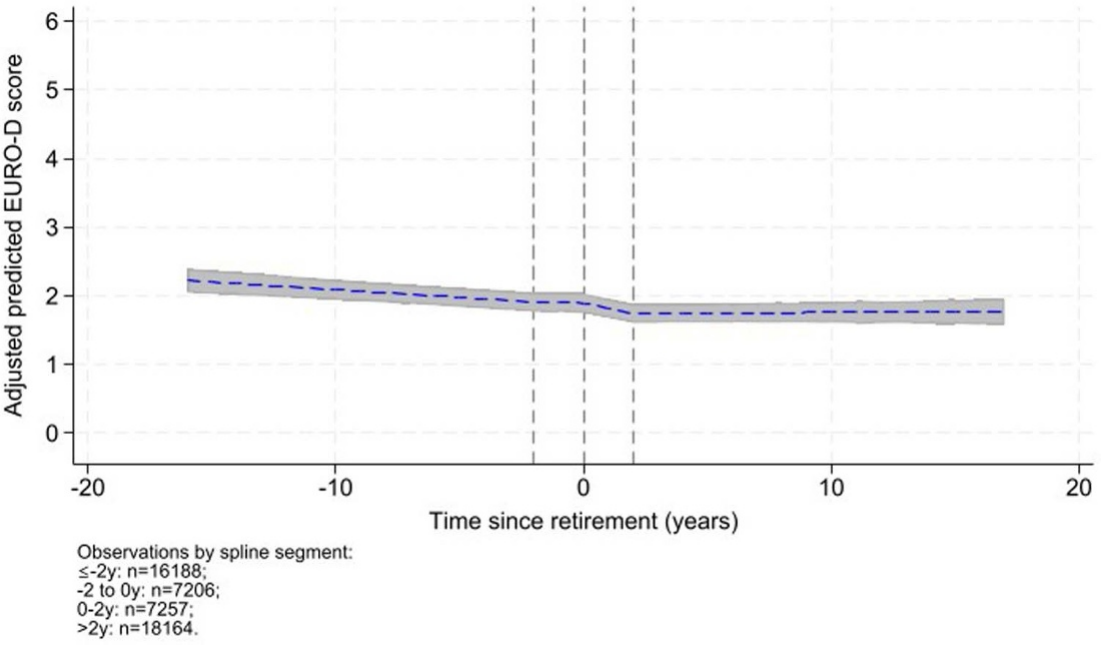
Adjusted and unweighted predicted values of EURO-D with 95% confidence intervals by time since retirement based on fixed effects from multilevel spline models from one imputation. Knot points at 2 years pre-, 0, and 2 years post-retirement. EURO-D scale ranges 0–12.

An initial multilevel model estimated 3.6% of between-country variation in EURO-D. We then include time since retirement (Table 2). We observed minimal changes in EURO-D scores in the period leading up to 2 years before retirement (0.01 points per year, 95% CI: −0.03 to 0.05) following adjustment for age, sex, years of education, household income, number of children and number of chronic conditions. Similarly, from 2 years before retirement until retirement, there was minimal additional change in EURO-D scores (−0.02 per year, 95% CI: −0.10 to 0.05). Starting at the time of retirement and up to 2 years post-retirement, EURO-D scores showed a slight decrease at an additional rate of −0.07 points per year (95% CI: −0.13, −0.0003). However, from 2 years post-retirement onward, EURO-D scores began to increase (0.09 points per year [95% CI: 0.02–0.16]). Figure 1 illustrates these results using the adjusted predicted values of EURO-D over time since retirement. Results for standardised EURO-D scores are in Supplementary Table S5 and Supplementary Figure S2.

**Table 2.** Multilevel spline models for associations between time since retirement and EURO-D Table 2 long description.

	*Empty model*	*Unadjusted*	*Adjusted*
*N*	10,698	10,698	10,698
**Intercept**			
Coefficient	1.873	1.926	2.647
95% CI	(1.702–2.045)	(1.708–2.144)	(1.167–4.126)
*p*-value	<0.001	<0.001	<0.001
**Years since retirement**			
Coefficient		0.003	0.011
95% CI		(−0.031 to 0.037)	(−0.028 to 0.051)
*p*-value		0.849	0.574
**Knot 1: <−2 years**			
Coefficient		−0.015	−0.020
95% CI		(−0.102 to 0.072)	(−0.095 to 0.054)
*p*-value		0.736	0.595
**Knot 2: 0 years**			
Coefficient		−0.062	−0.0652
95% CI		(−0.140 to 0.016)	(−0.1300 to 0.0003)
*p*-value		0.116	0.049
**Knot 3: >2 years**			
Coefficient		0.098	0.091
95% CI		(0.022–0.173)	(0.022–0.160)
*p*-value		0.011	0.010
**Individual random effect**			
Variance	1.156	0.245	0.213
95% CI	(0.982–1.361)	(0.099–0.608)	(0.084–0.536)
**Country random effect**			
Variance	0.357	0.353	0.276
95% CI	(0.255–0.499)	(0.252–0.495)	(0.216–0.351)

Years since retirement are centred at zero. Models are weighted by re-scaled calibrated survey weights at baseline and use imputed data.

Adjusted: age, sex, years of education, household income, number of children and number of chronic conditions.

**Figure 2.**
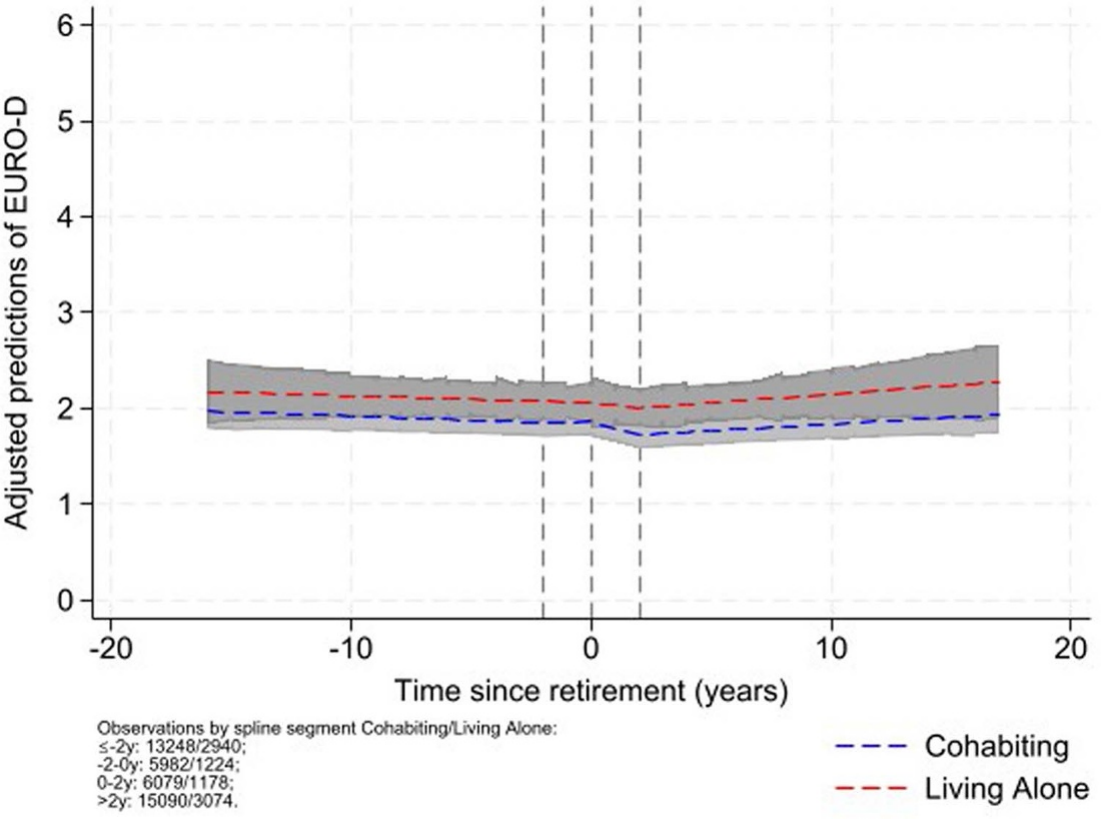
Adjusted and unweighted predicted values of EURO-D with 95% confidence intervals by time since retirement by cohabitation status based on fixed effects from multilevel spline models. Knot points at 2 years pre-, 0, and 2 years post-retirement. EURO-D scale ranges 0–12.

We found that those who lived alone at the time of retirement had consistently more depressive symptoms, i.e., a level difference. However, cohabitation status did not moderate the relationship between time since retirement and EURO-D (*p* = 0.107), and we did not observe differences when we stratified our analyses (Fig. 2 and Table S6). Results with standardised EURO-D are presented in Supplementary Table S7 and Figure S3. When stratified by sex, the decline in EURO-D scores around the time of retirement, followed by a subsequent increase, was observed particularly among males (*p* interaction = 0.002, Supplementary Table S8 and Supplementary Figure S4).

Supplementary Tables S9 and S10 and Supplementary Figures S5–S7 outline results from sensitivity analyses with alternative spline knot points. When we set the knot points to be at the time of retirement and 1 year pre- and post-retirement, we observe minimal changes in EURO-D scores in the periods leading up to 1 year before retirement and between 1 year before retirement and until retirement time. At the time of retirement, there was a small decline in EURO-D scores (−0.25 [95% CI: −0.44 to −0.05), followed by an increase starting 1 year post-retirement (0.22 [95% CI: 0.12–0.33]). When we set the knot points to be at the time of retirement and 6 years pre- and post-retirement, no changes in EURO-D scores were observed in the periods leading up to retirement or up to 6 years post-retirement. When the knot points were placed at the time of retirement and 10 years pre- and post-retirement, no changes in EURO-D scores were observed during any of the time periods.

### Research question two: what is the effect of retirement, as determined by statutory retirement age, on mental health and how does this effect differ based on cohabitation status?

We found that retirement was associated with a 0.16 lower score on EURO-D (95%CI: −0.21 to −0.11, i.e., better mental health) compared with employment (Supplementary Tables S11–S14) in our fixed-effect model.

Sex-, country- and year-specific statutory retirement age can be considered a good instrument. Of those eligible for retirement, 77% were retired (Supplementary Table S15). We observed a strong association between retirement eligibility and employment status (Supplementary Table S16), with a partial F-statistic of 1,570.54 and a sharp discontinuity in the proportion retired around the statutory retirement age (Supplementary Figure S8). Retirement eligibility was not associated with the confounders, conditional on age, sex and country (Supplementary Table S17) or with EURO-D conditional on employment status and confounders (Supplementary Table S18). We did not observe an association between retirement and depressive symptoms in our falsification test placebo test based on a statutory retirement age set 7 years earlier (Supplementary Table S19).

In our IV analyses, we found that retirement caused a 0.26 reduction (95%CI: −0.545 to −0.107) in EURO-D scores (Table 3 and S20). While we did not observe a statistically significant interaction term for retirement and cohabitation status (*p* = 0.840), indicating no evidence for effect modification, our stratified analyses showed that the relationship between retirement and better mental health was slightly stronger for those who cohabit than those who live alone when they retire. Supplementary Table S21 indicates that the results are consistent across the imputed datasets. We did not observe clear sex differences in our IV analysis (Supplementary Table S22, *p*-interaction = 0.557).

**Table 3.** Results from fixed-effect instrumental variable analyses

	Coefficient	95% CI	*p*-value
Full sample	−0.26	(−0.45 to −0.07)	0.008
Cohabitation	−0.27	(−0.48 to −0.06)	0.013
Living alone	−0.24	(−0.71 to 0.23)	0.315

Results based on observed data. Adjusted for age.

Cluster Robust SEs.

## Discussion

Our study aimed to address gaps in understanding the relationship between retirement and mental health by comparing individuals living alone with those cohabiting during the retirement transition and by applying a carefully triangulated methodological approach to overcome key methodological challenges. Overall, our study suggests that retirement leads to a slight improvement in depressive symptoms initially, but this effect does not appear to be sustained in the long term. This pattern appears strongest among men. While those who live alone during this transition consistently report more depressive symptoms than those who live with others, the impact of retirement does not seem to differ substantially between the groups.

Our study used triangulation to enhance reliability. The multilevel spline models examine associations between time since retirement and changes in depressive symptoms, but residual confounding may persist. Our IV approach, which used a robust instrument for retirement, minimised issues of confounding and reverse causation, but it is limited to assessing a LATE, i.e., the effect among those who retire because they reached statutory retirement age. Therefore, our findings may not apply to those who do not retire based on statutory retirement age. There have been previous empirical studies examining retirement and mental health. Our findings generally align with the conclusions of reviews of the topic (Odone *et al.*, 2021; van Ours, 2022). More specifically, our finding of short-term but not long-term effects of retirement on depressive symptoms using multilevel spline models is reassuringly consistent with other research that used SHARE data but applied different methods. Vo and Phu-Duyen found that retirement leads to an improvement in depressive symptoms shortly before and after retirement, but not in the long term (Vo and Phu-Duyen, 2023). Similarly, Mosconi *et al.* used generalised estimating equation models and found retirement was associated with a lower risk of depression in the short term, with a diminishing effect over time (Mosconi *et al.*, 2023). In addition to examining the dynamic longitudinal effect, by using multilevel models, we could explicitly model and account for the country-level differences in results.

Our IV analyses found that retirement results in a small improvement in depressive symptoms. Results from other studies applying an IV approach using statutory retirement age as an instrument have been mixed (Coe and Zamarro, 2011; Behncke, 2012; Belloni *et al.*, 2016; Heller-Sahlgren, 2017; Gorry *et al.*, 2018; Kolodziej and García-Gómez, 2019; Gorry and Slavov, 2021). For example, Kolodziej and García-Gómez (Kolodziej and García-Gómez, 2019) and Gorry *et al.* (Gorry *et al.*, 2018; Gorry and Slavov, 2021) find that, on average, retirement benefits mental health as measured by EURO-D and CES-D, respectively. However, Behncke (Behncke, 2012), who used self-reported doctor diagnosis as the mental health outcome, and Coe and Zamarro (Coe and Zamarro, 2011), who used EURO-D but restricted analyses to males only, did not observe relationships between retirement and mental health.

Identifying the average effect of retirement on depressive symptoms over the entire target population may not lead to the most meaningful insights. Previous studies have found that findings can vary by sex, marital status, occupational status, education and even during times of economic crisis (Eibich, 2015; Belloni *et al.*, 2016; Kolodziej and García-Gómez, 2019; Picchio and van Ours, 2020; Martinez Jimenez *et al.*, 2021). We were specifically interested in examining whether our results differed for those who cohabit compared with those who live alone during the transition from employment to retirement. It is plausible that those who live alone may be more vulnerable to mental health difficulties during the transition to retirement. Social isolation has been associated with poor mental health (Leigh-Hunt *et al.*, 2017), and retirement may lead to changes in social network (Börsch-Supan and Schuth, 2014; Comi *et al.*, 2022) that may affect the mental health of people who live alone differently. We found that individuals living alone reported more depressive symptoms, and while the IV estimate was stronger among those who cohabit, we did not find consistent evidence for differences in the association between retirement and mental health between the two groups. It is important to recognise the potential nuances within these subgroups that we did not capture, but future studies could explore. For example, individuals who live alone but maintain diverse social networks may be more protected against the negative effects of retirement compared to those who live alone with smaller social networks. Similarly, those who cohabit and are married may experience retirement differently from those who cohabit but remain single, and effects may vary by sex. For instance, Picchio and Van Ours, using participants from the Netherlands, find that for men who have a partner, retiring is good for their mental health, while there was no effect for partnered women (Picchio and van Ours, 2020). While broader aspects of social connectedness (e.g., network diversity, contact frequency and loneliness) may mediate the relationship between retirement and mental health differently for those living alone versus cohabiting, investigating these mechanisms requires a specific analytical framework and is beyond the scope of our study.

Key strengths of our study were the inclusion of participants from multiple European countries spanning 16 years, a triangulated approach and careful consideration for causality. Our analytical sample is younger, healthier and more socioeconomically advantaged than the excluded group (Supplementary Table S1). This reflects our focus on employment-to-retirement transitions. Therefore, our findings are most applicable to typical voluntary retirements between ages 50 and 75 and may not generalise to early or health-related retirements. We also analysed a straightforward transition to retirement from employment, examining its association with depressive symptoms. In reality, retirement may be more complex than this simple transition. Others have found differential effects on health outcomes depending on the type of transition (Dingemans and Henkens, 2015; Wels and Takami, 2021). Whether or not different retirement transitions affect the mental health of those who live alone compared with those who cohabit is also worth exploring in future studies. While our study examined cohabitation status, several other mechanisms may explain the observed trajectory of initial improvement followed by longer-term deterioration in depressive symptoms. Short-term benefits may reflect relief from work stress and increased leisure time, whereas longer-term increases could result from diminished social networks, loss of work identity or changes in health-related behaviours. Future research examining these mediating pathways would provide valuable insights into sustaining retirement’s mental health benefits.

Overall, we find that while retirement can initially alleviate depressive symptoms, this benefit is unlikely to be sustained in the long term. Individuals living alone consistently report more depressive symptoms, highlighting the importance of addressing social isolation in ageing populations. While our triangulated approach provides a thorough exploration of the topic, further work to examine more nuanced aspects of this transition would support the identification of the best interventions and strategies that promote mental well-being during this key life transition.

## Supporting information

10.1017/S2045796026100833.sm001 Maddock et al. supplementary material Maddock et al. supplementary material

## Data Availability

This paper uses data from SHARE Waves 1 (DOI:10.6103/SHARE.w1.900), 2 (10.6103/SHARE.w2.900), 4 (DOI:10.6103/SHARE.w4.900), 5 (DOI:10.6103/SHARE.w5.900), 6 (DOI:10.6103/SHARE.w6.900), 7 (DOI:10.6103/SHARE.w7.900) and 8 (DOI:10.6103/SHARE.w8.900) which is available at https://share-eric.eu/data/data-access.

## Supplementary material

The supplementary material for this article can be found at https://doi.org/10.1017/S2045796026100833.

## Table 1 Long description

Baseline characteristics at retirement are compared for participants who were cohabiting versus living alone, with totals also shown. The sample includes 10,698 people: 8,896 cohabiting (83 percent) and 1,802 living alone (17 percent). Average age is the same across groups at about 57 years. Gender differs notably: cohabiting participants are mostly male (61 percent), while those living alone are mostly female (60 percent). Depressive symptoms are higher among those living alone, with a mean EURO-D score of 2.37 compared with 1.88 for cohabiting participants. Years of education are very similar (about 12 years in both groups), and equivalised household income is slightly lower for those living alone (22,050 versus 23,451). Living alone is also associated with slightly more chronic conditions (1.17 versus 1.03) and fewer children in the household (1.44 versus 2.14). Values are weighted summary statistics and reflect observed data, so differences are descriptive rather than causal.

Navigate back to Table 1.

## Table 2 Long description

Multilevel spline model results relate time since retirement to EURO-D scores across three specifications: empty, unadjusted, and adjusted, each with 10,698 participants. Intercepts rise from about 1.87 in the empty model to about 2.65 in the adjusted model, with all intercepts statistically significant. The overall linear term for years since retirement is near zero and not statistically significant in both unadjusted and adjusted models. In the adjusted model, the spline segment at retirement (knot at 0 years) shows a small negative association and is borderline statistically significant, while the segment for more than 2 years since retirement shows a positive association and is statistically significant. The segment for less than minus 2 years is not statistically significant in either model. Random-effect variance is much larger for individuals than for countries, and both variances are lower in the unadjusted and adjusted models than in the empty model, indicating reduced unexplained variability after adding predictors.

Navigate back to Table 2.
