# Supplementary material for: Retirement timing, living arrangements and mental health: a multi-method investigation using Survey of Health, Ageing and Retirement in Europe
Source: Epidemiol Psychiatr Sci. 2026 Aug 5;35:e47. doi: 10.1017/S2045796026100833 (PMC13444393; doi:10.1017/S2045796026100833)
Supplement: Supplementary file 1 [file S2045796026100833sup001.pdf]

Supplementary Materials

Retirement timing, cohabitation status, and mental health: a multi-method investigation using Survey of Health, Ageing and Retirement in Europe (2004-2020)

J Maddock[1,5], C Simmons[2, 3], J Wels[4, 5], P Patalay[5, 6]

[1]Division of Surgery and Interventional Science, Institute of Sport, Exercise and Health, University College London, London, UK.

[2]Health & Care, European Centre for Social Welfare Policy and Research, Vienna, Austria.

[3]Erasmus School of Health Policy and Management, Erasmus University Rotterdam, Rotterdam, the Netherlands

[4]Université libre de Bruxelles, Brussels, Belgium.

[5]Unit for Lifelong Health and Ageing at UCL, University College London, London, UK.

[6]Centre for Longitudinal Studies, UCL Social Research Institute, University College London, London, UK.

Chronic conditions included:

A heart attack including myocardial infarction or coronary thrombosis or any other heart problem including congestive heart failure; High blood pressure or hypertension; High cholesterol; A stroke or cerebral vascular disease; Diabetes or high blood sugar; Chronic lung disease such as chronic bronchitis or emphysema; Asthma; Arthritis, including osteoarthritis, or rheumatism; Osteoporosis; Cancer or malignant tumour, including leukaemia or lymphoma, but excluding minor skin cancers; Stomach or duodenal ulcer, peptic ulcer; Cataracts; Hip fracture or femoral fracture or other condition.

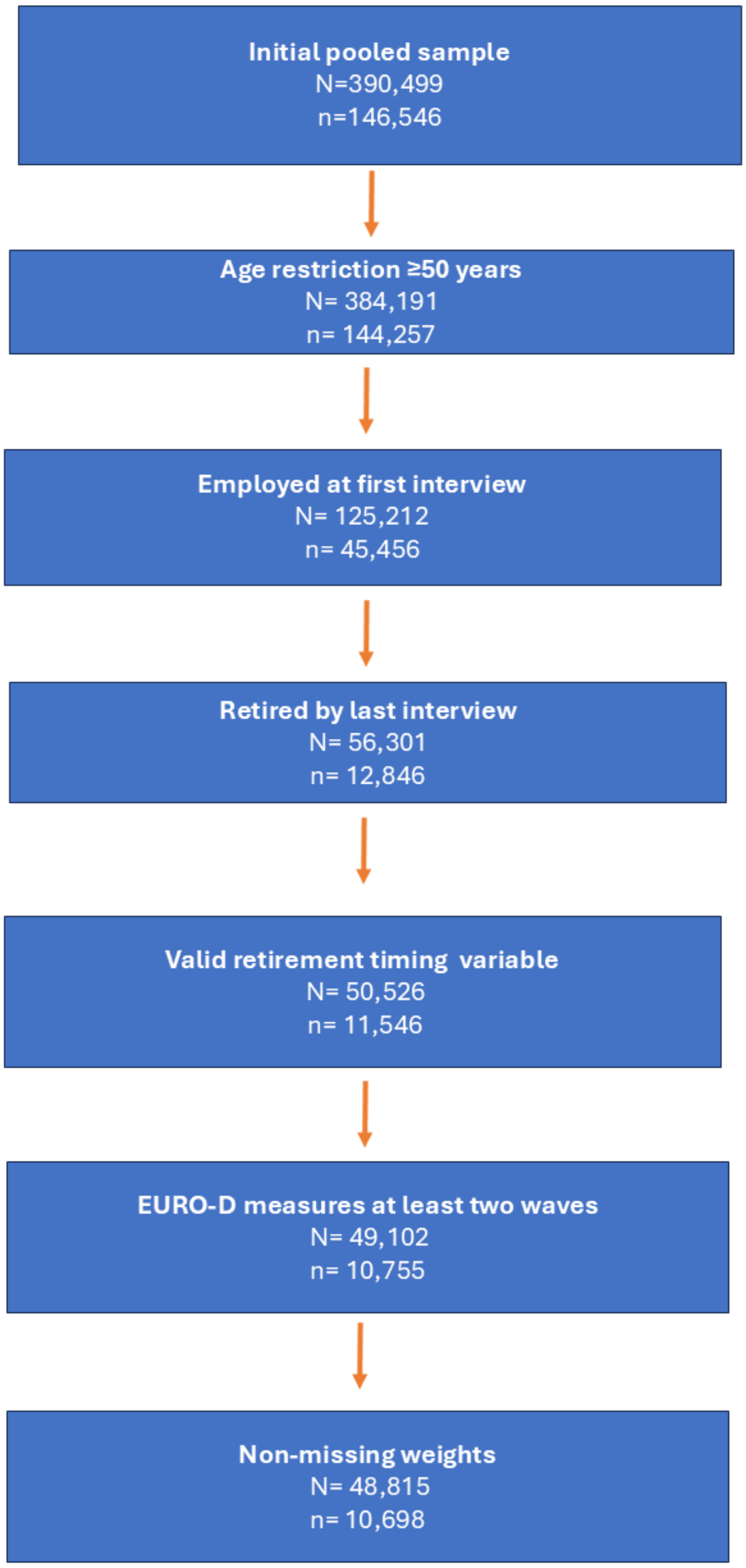

Figure S1. Sample selection from SHARE waves 1-8 (2004-2020)

Analytical sample from SHARE waves 1-8, Release 9.0.0. SHARE's multiple imputation used for missing data. Sample restricted to individuals aged 50+, employed at baseline (observed data), retired by final interview (observed data), with valid retirement timing and ≥2 EURO-D measurements over time.

Table S1: Comparison of baseline characteristics between excluded SHARE sample and included analytical sample after restrictions as outlined in Figure S1

	**SHARE (excluded)**	**Analytical sample**
	135,848	10,698
**Age (years**)	64.7 (10.7)	58.0 (4.7)
**Gender**		
Male	59,373 (44%)	5,667 (53%)
Female	76,475 (56%)	5,031 (47%)
**EURO-D**	2.52 (2.33)	1.91 (1.89)
**Years of education**	10.63 (4.30)	12.10 (4.08)
**Equivalised household income**	17,475 (25,221)	27,069 (31,272)
**Number of chronic diseases**	1.75 (1.60)	1.11 (1.15)
**Cohabitation Status**		
Not Alone	62,657 (77%)	8,896 (83%)
Alone	18,793 (23%)	1,802 (17%)

Analytical sample restricted to those employed at baseline, retired by last interview, valid retirement timing, ≥2 EURO-D measurements, and non-missing weights. Values are mean (SD) or N (%). EURO-D ranges 0-12 (higher=more symptoms).

Table S2: Distribution of observations over time

N	10,698
Number of times observed	
2	837 (8%)
3	1,719 (16%)
4	2,634 (25%)
5	2,798 (26%)
6	1,512 (14%)
7	1,198 (11%)

Table S3: Distribution of participants and observations by country

	N individuals	Observations
Country of residence		
Austria	543	11197
Germany	768	18696
Sweden	1073	29253
Netherlands	544	7740
Spain	591	13710
Italy	495	12558
France	973	25677
Denmark	799	23143
Greece	569	9495
Switzerland	695	18980
Belgium	873	23458
Israel	419	9201
Czech Republic	778	16058
Poland	228	4039
Luxembourg	148	1836
Hungary	113	952
Portugal	52	398
Slovenia	289	5125
Estonia	691	12043
Croatia	57	488

Table S4: EURO-D at baseline by sex and cohabitation status

	**Cohabiting**		**Living Alone**		**Total**	
	Mean	95% CI	Mean	95% CI	Mean	95% CI
Male	1.57	(1.50,1.65)	1.88	(1.62,2.16)	1.61	(1.54,1.69)
Female	2.40	(2.30,2.51)	2.69	(2.47,2.91)	2.49	(2.39,2.59)

Values are weighted using re-scaled calibrated survey weights at baseline and use imputed data. EURO-D ranges from 0-12 where higher scores indicate more depressive symptoms

Table S5: Multilevel spline models showing associations between time since retirement and standardised EURO-D

	Empty model	Unadjusted	Adjusted
**N**	10,698	10,698	10,698
**Intercept**			
Coefficient	0.020	0.048	0.431
95% CI	(-0.072,0.111)	(-0.068,0.164)	(-0.359,1.221)
p-value	0.672	0.420	0.285
**Years since retirement**			
Coefficient		0.002	0.006
95% CI		(-0.016,0.020)	(-0.015,0.027)
p-value		0.846	0.573
**Knot 1: <-2 years**			
Coefficient		-0.008	-0.011
95% CI		(-0.055,0.039)	(-0.051,0.029)
p-value		0.736	0.595
**Knot 2: 0 years**			
Coefficient		-0.033	-0.035
95% CI		(-0.075,0.008)	(-0.070, -0.000)
p-value		0.117	0.049
**Knot 3: >2 years**			
Coefficient		0.052	0.049
95% CI		(0.012,0.092)	(0.012,0.085)
p-value		0.011	0.009
**Individual random effect**			
Coefficient	0.618	0.131	0.114
95% CI	(0.525,0.727)	(0.053,0.325)	(0.045,0.287)
**Country random effect**			
Variance	0.190	0.189	0.147
95% CI	(0.136,0.266)	(0.135,0.264)	(0.115,0.188)

Years since retirement is centred at zero. Models are weighted by re-scaled calibrated survey weights at baseline and use imputed data.
Adjusted: age, sex, years of education, household income, number of children, number of chronic conditions. EURO-D standardised to 0 mean 1 SD

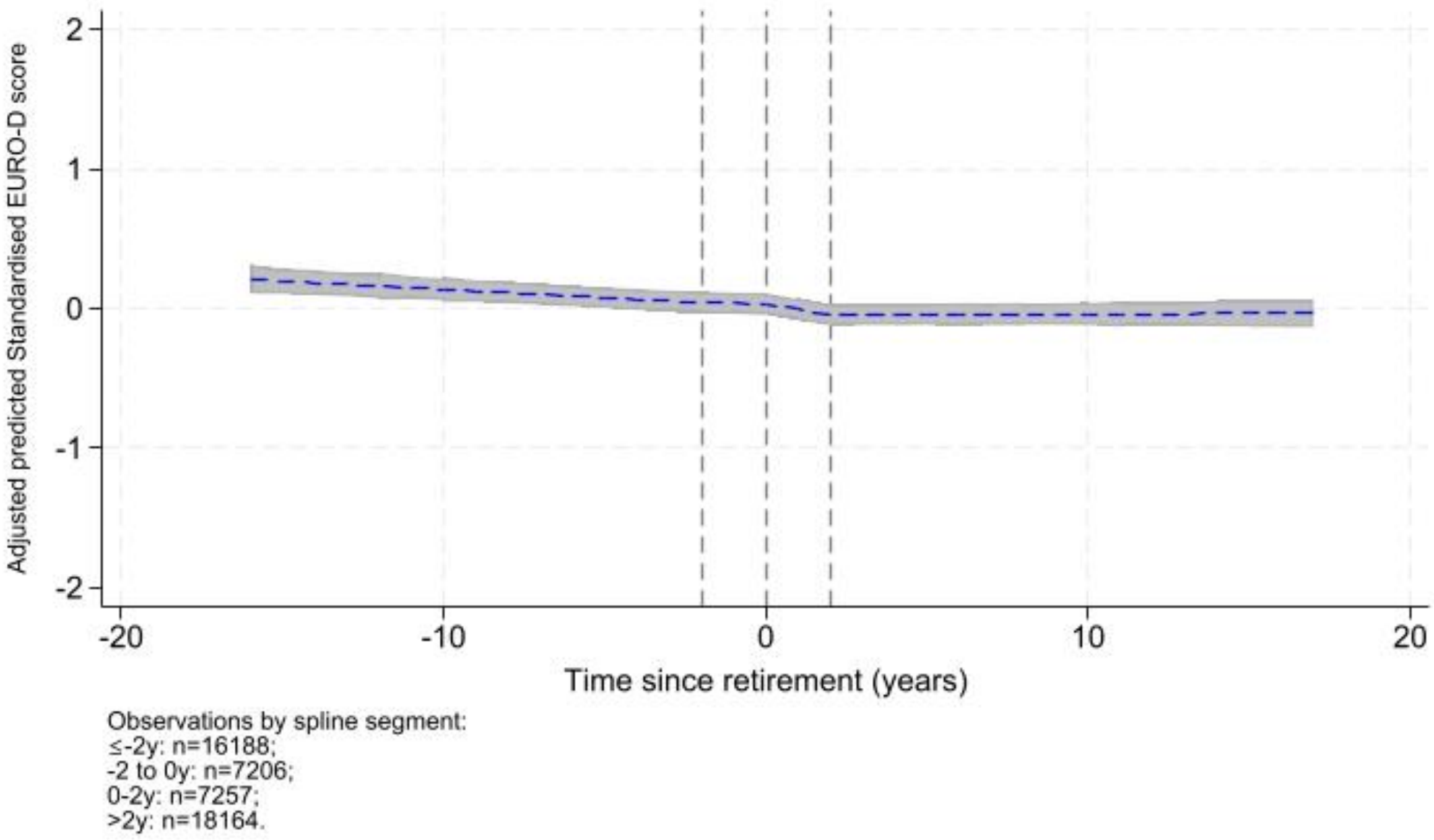

Figure S2: Adjusted unweighted predicted values of standardised EURO-D with 95% confidence intervals by time since retirement based on fixed effects from multilevel spline models based on one imputation. Knot points at 2 years pre-, 0, and 2 years post-retirement

Table S6: Multilevel spline models for associations between time since retirement and EURO-D stratified by cohabitation status at the time of retirement

	Cohabiting	Living alone
**N**	8,896	1,802
**Intercept**		
Coefficient	2.741	3.164
95% CI	(1.486,3.996)	(-0.195,6.522)
p-value	0.000	0.065
**Years since retirement**		
Coefficient	0.011	0.031
95% CI	(-0.023,0.046)	(-0.048,0.110)
p-value	0.519	0.438
**Knot 1: <-2 years**		
Coefficient	-0.001	-0.103
95% CI	(-0.079,0.076)	(-0.248,0.042)
p-value	0.970	0.163
**Knot 2: 0 years**		
Coefficient	-0.085	0.017
95% CI	(-0.182,0.013)	(-0.138,0.172)
p-value	0.089	0.831
**Knot 3: >2 years**		
Coefficient	0.095	0.074
95% CI	(0.003,0.187)	(-0.007,0.156)
p-value	0.042	0.073
**Individual random effect**		
Variance	0.190	0.315
95% CI	(0.078,0.464)	(0.103,0.965)
**Country random effect**		
Variance	0.275	0.345
95% CI	(0.212,0.357)	(0.223,0.535)

Years since retirement is centred at zero. Models are weighted by re-scaled calibrated survey weights at baseline and use imputed data. Adjusted: age, sex, years of education, household income, number of children, number of chronic conditions.

Table S7 Multilevel spline models for associations between time since retirement and standardised EURO-D stratified by cohabitation status at the time of retirement

	**Cohabiting**	**Living alone**
**N**	8,896	1,802
**Intercept**		
Coefficient	0.481	0.707
95% CI	(-0.189,1.151)	(-1.087,2.501)
p-value	0.159	0.440
**Years since retirement**		
Coefficient	0.006	0.017
95% CI	(-0.013,0.025)	(-0.025,0.059)
p-value	0.519	0.437
**Knot 1: <-2 years**		
Coefficient	-0.001	-0.055
95% CI	(-0.042,0.040)	(-0.133,0.022)
p-value	0.970	0.163
**Knot 2: 0 years**		
Coefficient	-0.045	0.009
95% CI	(-0.097,0.007)	(-0.074,0.092)
p-value	0.089	0.833
**Knot 3: >2 years**		
Coefficient	0.051	0.040
95% CI	(0.002,0.100)	(-0.004,0.083)
p-value	0.042	0.073
**Individual random effect**		
Variance	0.102	0.168
95% CI	(0.042,0.248)	(0.055,0.516)
**Country random effect**		
Variance	0.147	0.185
95% CI	(0.113,0.190)	(0.119,0.286)

Years since retirement is centred at zero. Models are weighted by re-scaled calibrated survey weights at baseline and use imputed data. Adjusted: age, sex, years of education, household income, number of children, number of chronic conditions. EURO-D standardised to 0 mean 1 SD.

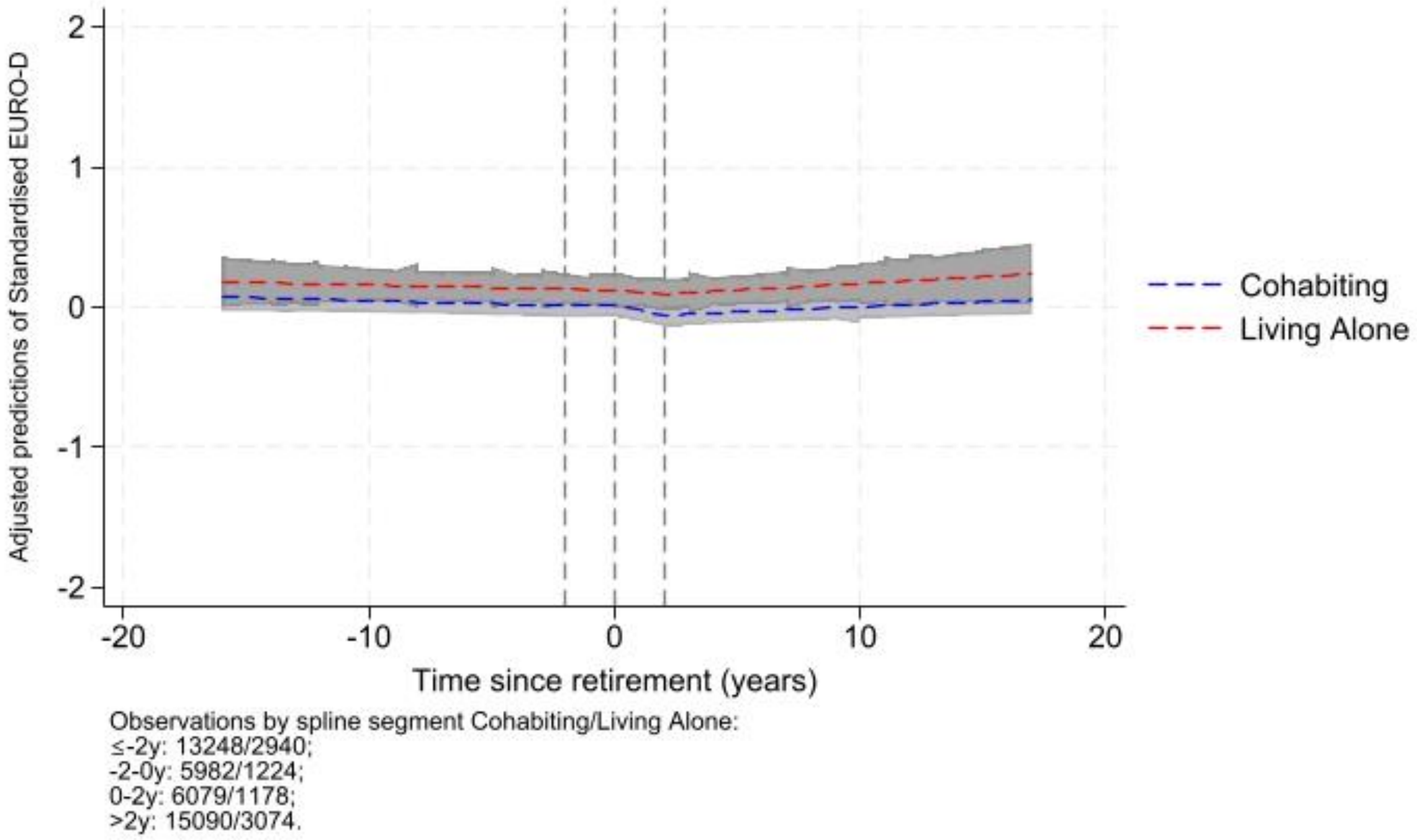

Figure S3: Adjusted unweighted predicted values of standardised EURO-D with 95% confidence intervals by time since retirement by cohabitation status based on fixed effects from multilevel spline models and one imputation. Knot points at 2 years pre-, 0, and 2 years post-retirement

Table S8 Multilevel spline models for associations between time since retirement and EURO-D stratified by sex

	Male	Female
**N**	5,677	5,031
**Intercept**		
Coefficient	1.530	5.174
95% CI	(-0.094,3.155)	(3.695,6.652)
p-value	0.065	<0.0001
**Years since retirement**		
Coefficient	0.011	0.020
95% CI	(-0.019,0.041)	(-0.038,0.079)
p-value	0.472	0.497
**Knot 1: <-2 years**		
Coefficient	-0.014	-0.026
95% CI	(-0.077,0.050)	(-0.126,0.074)
p-value	0.677	0.606
**Knot 2: 0 years**		
Coefficient	-0.134	0.020
95% CI	(-0.207,-0.060)	(-0.055,0.095)
p-value	<0.0001	0.602
**Knot 3: >2 years**		
Coefficient	0.160	0.004
95% CI	(0.091,0.230)	(-0.083,0.091)
p-value	<0.0001	0.923
**Individual random effect**		
Variance	0.206	0.211
95% CI	(0.079,0.537)	(0.084,0.530)
**Country random effect**		
Variance	0.239	0.337
95% CI	(0.175,0.328)	(0.262,0.435)

Years since retirement is centred at zero. Models are weighted by re-scaled calibrated survey weights at baseline and use imputed data. Adjusted: age, years of education, household income, number of children, number of chronic conditions, cohabitation status.

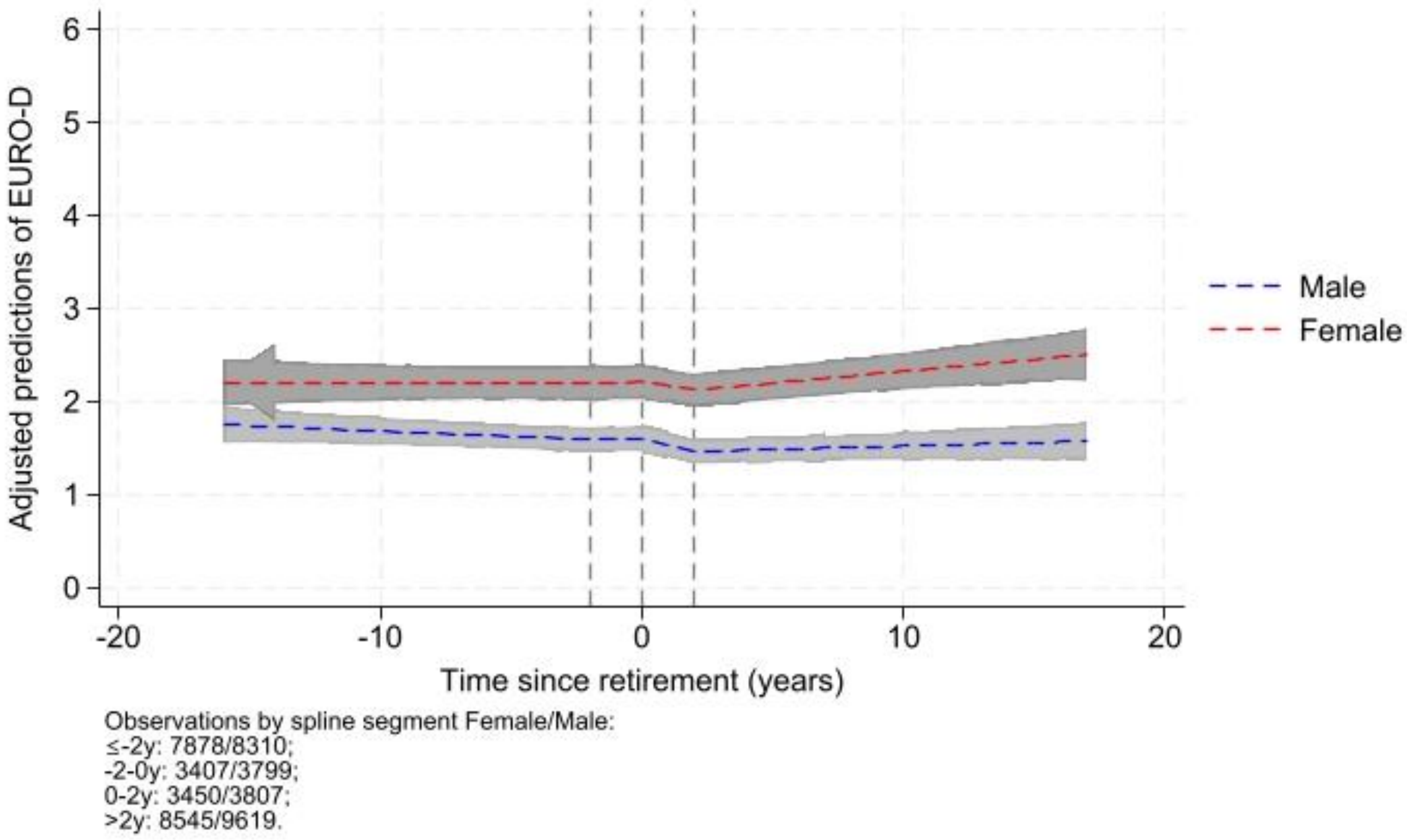

Figure S4: Adjusted unweighted predicted values of EURO-D with 95% confidence intervals by time since retirement by sex based on fixed effects from multilevel spline models and one imputation. Knot points at 2 years pre-, 0, and 2 years post-retirement. EURO-D scale ranges 0-12

Table S9: Sensitivity analyses with different spline knot points

	Splines at ±1 year	*Splines at ± 6 year*	*Splines at ±10 years*
N	10,698	10,698	10,698
**Intercept**			
Coefficient	2.54	2.67	3.26
95% CI	(1.07,4.02)	(1.47,3.87)	(1.60,4.91)
p-value	<0.001	<0.001	<0.001
**Years since retirement**			
Coefficient	0.01	0.03	0.08
95% CI	(-0.03,0.04)	(-0.01,0.06)	(-0.08,0.24)
p-value	0.75	0.19	0.32
**Knot 1 (time prior to retirement)**			
Coefficient	0.03	-0.03	-0.09
95% CI	(-0.13,0.19)	(-0.10,0.03)	(-0.27,0.09)
p-value	0.70	0.32	0.33
**Knot 2: 0 years**			
Coefficient	-0.25	-0.01	0.00
95% CI	(-0.44,-0.05)	(-0.05,0.04)	(-0.03,0.04)
p-value	0.01	0.79	0.84
**Knot 3 (time post retirement)**			
Coefficient	0.22	0.04	0.05
95% CI	(0.12,0.33)	(-0.00,0.08)	(-0.02,0.12)
p-value	0.00	0.07	0.13
**Individual random effect**			
Coefficient	0.19	0.16	0.11
95% CI	(0.07,0.48)	(0.10,0.25)	(0.08,0.16)
**Country random effect**			
Coefficient	0.28	0.29	0.30
95% CI	(0.22,0.35)	(0.22,0.39)	(0.22,0.40)

Years since retirement is centred at zero. Models are weighted by re-scaled calibrated survey weights at baseline and use imputed data. Adjusted: age, sex, years of education, household income, number of children, number of chronic conditions.

Table S10: Sensitivity analyses with different spline knot points stratified by cohabitation status

	Cohabiting	Living alone	Cohabiting	Living alone	Cohabiting	Living alone
	Splines at ±1 year		*Splines at ± 6 year*		*Splines at ±10 years*	
N	8,951	1,802	8,951	1,802	8,951	1,802
**Intercept**						
Coefficient	2.65	2.83	2.87	2.75	3.40	3.68
95% CI	(1.37,3.93)	(-0.57,6.22)	(1.76,3.97)	(-0.08,5.57)	(1.13,5.66)	(1.39,5.98)
p-value	0.00	0.10	0.00	0.06	0.00	0.00
**Years since retirement**						
Coefficient	0.01	0.02	0.03	0.01	0.09	0.08
95% CI	(-0.02,0.04)	(-0.06,0.09)	(-0.03,0.10)	(-0.07,0.08)	(-0.12,0.30)	(0.01,0.16)
p-value	0.59	0.70	0.28	0.88	0.42	0.03
**Knot 1 (time prior to retirement)**						
Coefficient	0.06	-0.13	-0.04	-0.02	-0.09	-0.10
95% CI	(-0.08,0.20)	(-0.39,0.13)	(-0.14,0.06)	(-0.08,0.04)	(-0.32,0.14)	(-0.19,-0.01)
p-value	0.39	0.33	0.41	0.57	0.43	0.03
**Knot 2: 0 years**						
Coefficient	-0.29	-0.01	-0.01	-0.01	0.00	0.01
95% CI	(-0.48,-0.10)	(-0.38,0.36)	(-0.06,0.05)	(-0.05,0.03)	(-0.03,0.03)	(-0.03,0.05)
p-value	0.00	0.94	0.84	0.59	0.93	0.63
**Knot 3 (time post retirement)**						
Coefficient	0.24	0.14	0.04	0.04	0.05	0.05
95% CI	(0.12,0.36)	(-0.08,0.36)	(-0.01,0.09)	(0.00,0.07)	(-0.01,0.12)	(-0.08,0.17)
p-value	0.00	0.22	0.13	0.03	0.12	0.47
**Individual random effect**						
Coefficient	0.17	0.29	0.15	0.20	0.11	0.14
95% CI	(0.07,0.40)	(0.05,1.88)	(0.10,0.24)	(0.12,0.32)	(0.07,0.15)	(0.09,0.20)
**Country random effect**						
Coefficient	0.28	0.35	0.30	0.30	0.30	0.30
95% CI	(0.21,0.36)	(0.24,0.53)	(0.22,0.41)	(0.21,0.42)	(0.22,0.41)	(0.21,0.41)

Years since retirement is centred at zero. Models are weighted by re-scaled calibrated survey weights at baseline and use imputed data. Adjusted: age, sex, years of education, household income, number of children, number of chronic conditions.

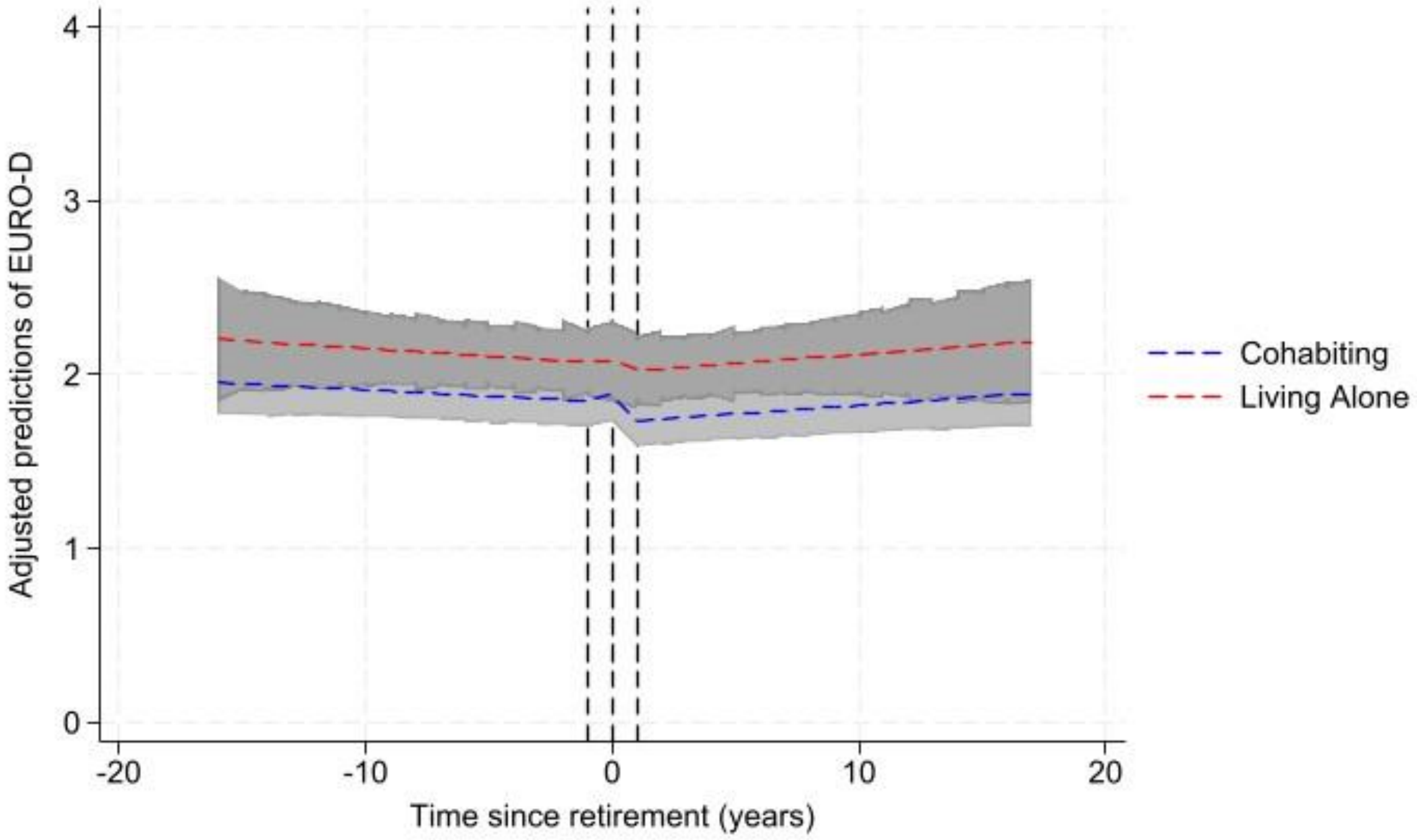

Figure S5: Adjusted unweighted predicted values of EURO-D with 95% confidence intervals by time since retirement by cohabitation status based on fixed effects from multilevel spline models from one imputation. Knot points at 1 year pre-, 0, and 1 year post-retirement. EURO-D scale ranges 0-12.

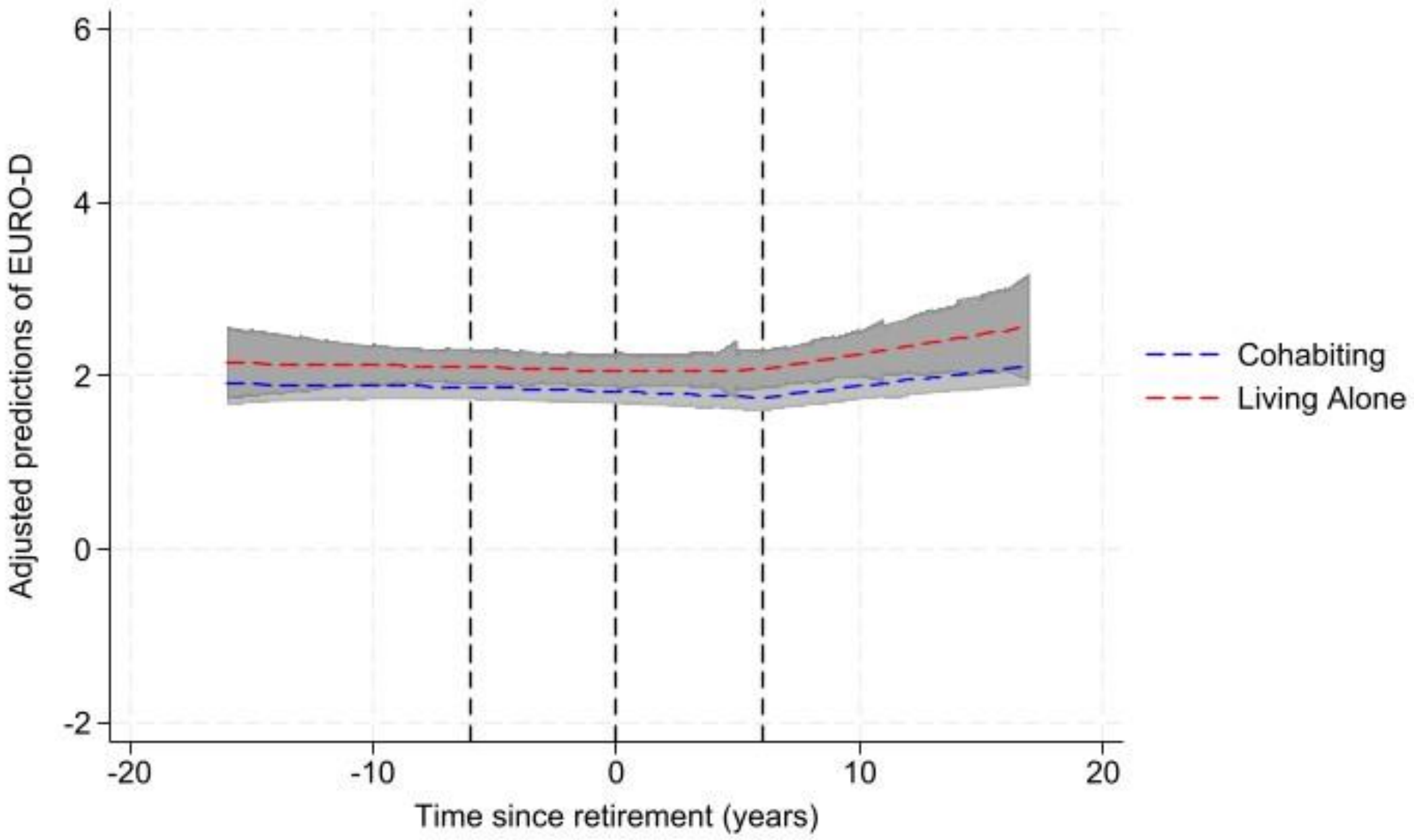

Figure S6: Adjusted unweighted predicted values of EURO-D with 95% confidence intervals by time since retirement by cohabitation status based on fixed effects from multilevel spline models. Knot points at 6 years pre-, 0, and 6 years post-retirement.EURO-D scale ranges 0-12.

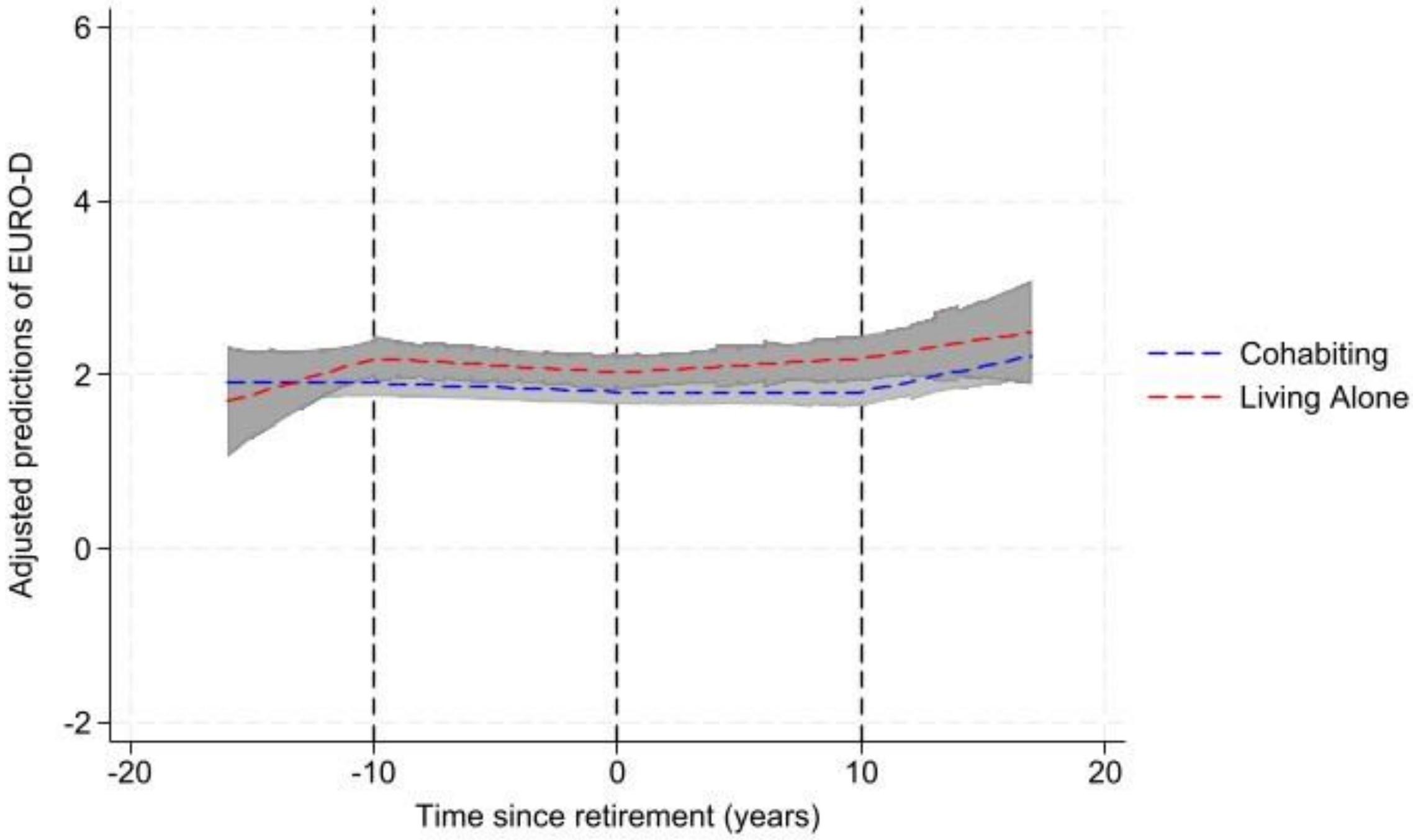

Figure S7: Adjusted unweighted predicted values of EURO-D with 95% confidence intervals by time since retirement by cohabitation status based on fixed effects from multilevel spline models. Knot points at 10 years pre-, 0, and 10 years post-retirement. EURO-D scale ranges 0-12.

Table S11 Fixed-effect model of retirement status and EURO-D

**N individuals**	10,698
**Retired**	
Coefficient	-0.157
95% CI	(-0.207,-0.106)
p-value	0.000
**Intercept**	
Coefficient	1.816
95% CI	(1.421,2.211)
p-value	0.000
**Panel-level standard deviation**	1.408
**Standard deviation of ε_it**	1.410
ρ	0.499

Results from one imputed dataset (mi estimate not compatible with Stata xtreg (see complete case in Table S12 for comparison). Models adjusted for age, years of education, household income, number of children, number of chronic conditions. Cluster robust SEs

Table S12 Fixed-effect model of retirement status and EURO-D (complete case)

**N individuals**	7,645
**Retired**	
Coefficient	-0.191
95% CI	(-0.291,-0.092)
p-value	<0.0001
**Intercept**	
Coefficient	1.372
95% CI	(0.589,2.154)
p-value	0.001
**Panel-level standard deviation**	1.638
**Standard deviation of ε_it**	1.381
ρ	0.584

Results from complete cases (mi estimate not compatible with Stata xtreg (see results from Table S11 for comparison). Models adjusted for age, years of education, household income, number of children, number of chronic conditions. Cluster robust SEs

Table S13 Fixed-effect model of retirement status and standardised EURO-D

	10,698
**Retired**	
Coefficient	-0.157
95% CI	(-0.207,-0.106)
p-value	<0.0001
**Intercept**	
Coefficient	1.816
95% CI	(1.421,2.211)
p-value	0.000
**Panel-level standard deviation**	1.408
**Standard deviation of ε_it**	1.410
ρ	0.499

Results from one imputed dataset (mi estimate not compatible with Stata xtreg (see complete case in Table S14 for comparison). Models adjusted for age, years of education, household income, number of children, number of chronic conditions. Cluster robust SEs

Table S14 Fixed-effect model of retirement status and standardised EURO-D (complete case)

	7,645
**Retired**	
Coefficient	-0.102
95% CI	(-0.156,-0.049)
p-value	<0.0001
**Intercept**	
Coefficient	-0.247
95% CI	(-0.666,0.172)
p-value	0.248
**Panel-level standard deviation**	0.876
**Standard deviation of ε_it**	0.739
**ρ**	0.584

Results from complete cases (mi estimate not compatible with Stata xtreg (see results from Table S13 for comparison). Models adjusted for age, years of education, household income, number of children, number of chronic conditions. Cluster robust SEs

Table S15: Distribution of employment status and retirement eligibility

	Employment status	
	Employed	Retired
	21,249	25,994
**Retirement Eligibility**		
Not eligible	17,851 (84%)	5,864 (23%)
Eligible	3,398 (16%)	20,130 (77%)

Values are unweighted and from observed data. Numbers reflect total observations, not individuals.

Table S16: Association between retirement and retirement eligibility

**Retirement Eligibility**	
Coefficient	14.637
95% CI	(13.924,15.386)
p-value	<0.0001

Results from logistic regression, odds ratio using observed data
Cluster robust SEs

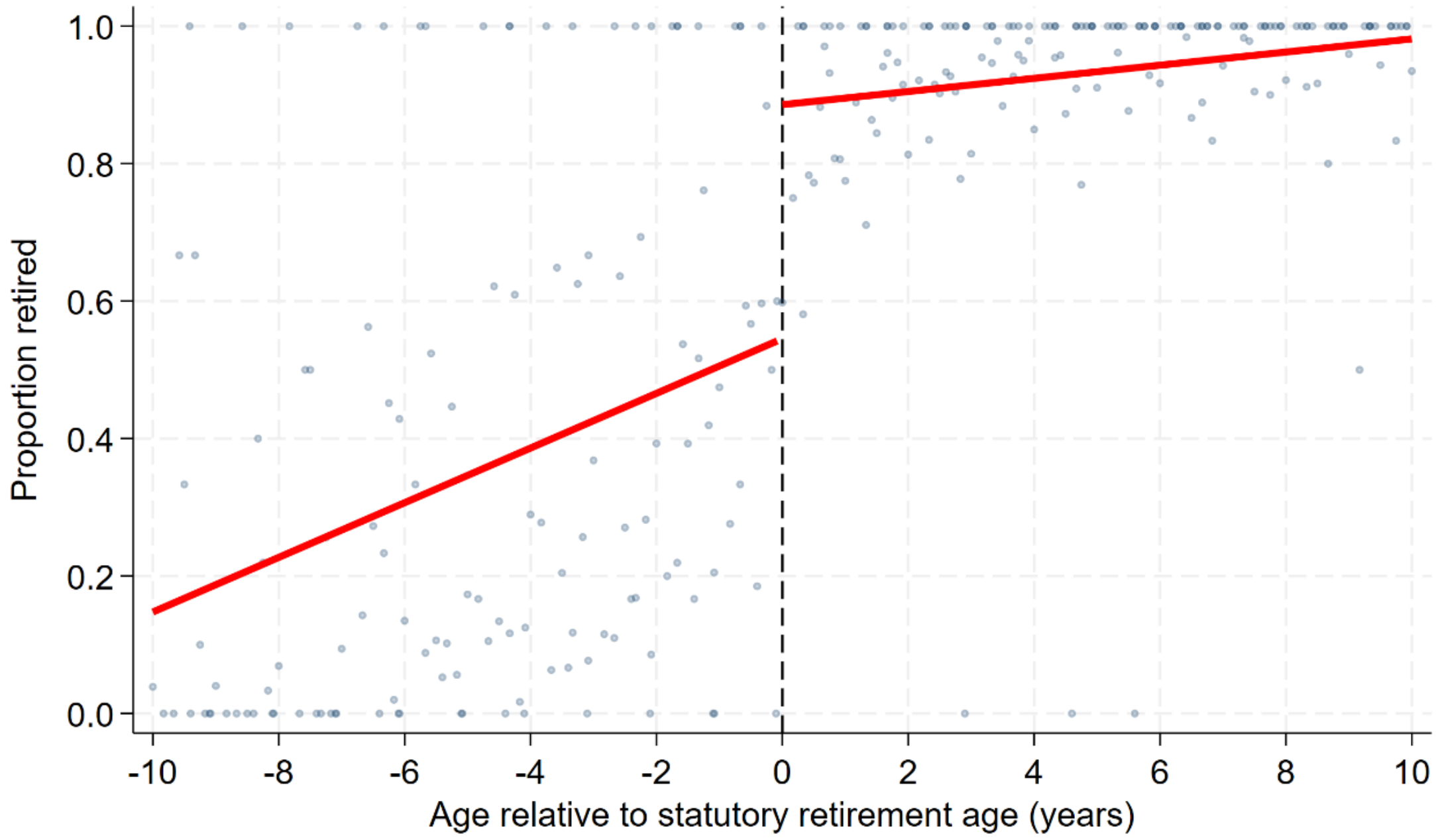

Figure S8: Proportion of sample retired by age relative to statutory retirement age using observed data. Red lines show linear fits above and below the eligibility threshold.

Table S17: Association between confounders and retirement eligibility or employment status

	Retirement eligibility				Employment status			
	F statistic	Coefficient	95% CI	p-value	F statistic	Coefficient	95% CI	p-value
Years of education	54.85	0.034	(-0.079,0.147)	0.555	57.54	-0.402	(-0.529,-0.274)	<0.0001
Equivalised household income	410.06	-500	(-1700 ,745)	0.431	738.07	-2705	(-3520, -1890)	<0.0001
Number of children	18.99	0.006	(-0.029,0.041)	0.731	19.32	-0.048	(-0.086,-0.010)	0.014
Number of chronic conditions	53.99	-0.005	(-0.042,0.033)	0.810	69.56	0.151	(0.113,0.188)	<0.0001
Reasons for retirement	59.30	-0.010	(-0.038,0.017)	0.474	60.29	0.028	(-0.001,0.058)	0.061

Estimates from observed data adjusted for age, sex and country. Cluster robust SEs

Table S18: Associations of retirement eligibility with EURO-D conditional on employment status and confounders

Coefficient	95% CI	p-value
0.016	(-0.040,0.072)	0.571

Estimates from observed data. Adjusted for employment status, age, sex, years of education, household income, number of children, number of chronic conditions, country of residence. Cluster robust SEs

Table S19: Falsification test using fixed-effect instrumental variable

	Coefficient	95% CI	p-value
Full sample	0.424	(-2.285,3.134)	0.759
Cohabiting	1.213	(-2.046,4.471)	0.466
Living alone	-3.035	(-10.529,4.458)	0.427

Estimates from observed data

Table S20. Results from fixed-effect instrumental variable analyses for standardised EURO-D

	Coefficient	95% CI	p-value
Full sample	-0.140	(-0.243,-0.037)	0.008
Cohabitation	-0.143	(-0.256,-0.031)	0.013
Alone	-0.128	(-0.377,0.121)	0.315

Results based on observed data. Adjusted for age
Cluster Robust SEs

Table S21. Results from fixed-effect instrumental variable analyses for EURO-D across imputations

	Imputation 1	Imputation 2	Imputation 3	Imputation 4
	Coefficient (95% CI)	Coefficient (95% CI)	Coefficient (95% CI)	Coefficient (95% CI
Full sample	-0.258 (-0.452,-0.065)	-0.252 (-0.444,-0.060)	-0.279 (-0.472,-0.085)	-0.258 (-0.450,-0.066)
Cohabitation	-0.260 (-0.473,-0.047)	-0.246 (-0.457,-0.035	-0.278 (-0.491,-0.066)	-0.259 (-0.470,-0.048)
Alone	-0.256 (-0.719,0.207)	-0.280 (-0.744,0.184)	-0.286 (-0.752,0.179)	-0.259 (-0.721,0.203)

Results based on observed data. Adjusted for age
Cluster Robust SEs. Numbers represent different imputed datasets.

Table S22. Results from fixed-effect instrumental variable analyses for EURO-D stratified by sex

	Coefficient	95% CI	p-value
Male	-0.218	(-0.471, 0.035)	0.091
Female	-0.304	(-0.594, -0.014)	0.040

Results based on observed data. Adjusted for age
Cluster Robust SEs
